# The Burden of Neonatal Invasive Candidiasis in Low- and Middle-income Countries: A Systematic Review and Meta-analysis

**DOI:** 10.1093/ofid/ofaf329

**Published:** 2025-06-11

**Authors:** Daniel Hsiang-Te Tsai, Ian Chang-Yen Wu, Ling-Fang Chang, Marie Jen-Huey Lu, Brishti Debnath, Nelesh P Govender, Mike Sharland, Adilia Warris, Yingfen Hsia, Laura Ferreras-Antolin

**Affiliations:** Centre for Neonatal and Paediatric Infection, City St. George's, University of London, London, UK; School of Pharmacy, Institute of Clinical Pharmacy and Pharmaceutical Sciences, College of Medicine, National Cheng Kung University, Tainan, Taiwan; Centre for Neonatal and Paediatric Infection, City St. George's, University of London, London, UK; School of Pharmacy, Institute of Clinical Pharmacy and Pharmaceutical Sciences, College of Medicine, National Cheng Kung University, Tainan, Taiwan; Department of Pharmacy, National Cheng Kung University Hospital, College of Medicine, National Cheng Kung University, Tainan, Taiwan; Institute of Public Health, School of Medicine, National Yang-Ming Chiao Tung University, Taipei, Taiwan; Department of Pharmacy, Taipei Veterans General Hospital, Taipei, Taiwan; Department of Pharmacy, Colleage of Pharmaceutical Sciences, National Yang Ming Chiao Tung University, Taipei, Taiwan; Centre for Neonatal and Paediatric Infection, City St. George's, University of London, London, UK; Wits Mycology Division, Faculty of Health Sciences, University of the Witwatersrand, Johannesburg, South Africa; Institute for Infection and Immunity, City St. George's, University of London, London, UK; Medical Research Council Centre for Medical Mycology, University of Exeter, Exeter, UK; Centre for Neonatal and Paediatric Infection, City St. George's, University of London, London, UK; Medical Research Council Centre for Medical Mycology, University of Exeter, Exeter, UK; Centre for Neonatal and Paediatric Infection, City St. George's, University of London, London, UK; Acute Respiratory Unit, UK Health Security Agency, London, UK; Centre for Neonatal and Paediatric Infection, City St. George's, University of London, London, UK; Medical Research Council Centre for Medical Mycology, University of Exeter, Exeter, UK

**Keywords:** candidemia, case fatality rate, incidence, low- and middle-income countries, neonatal invasive candidiasis

## Abstract

**Background:**

Invasive *Candida* infection remains a significant threat to neonates worldwide. Most evidence on neonatal invasive candidiasis (NIC) comes from high-income countries, leaving the burden and characteristics of NIC in low- and middle-income countries (LMICs) poorly described. This study aimed to investigate the incidence, case-fatality rates (CFR), epidemiology, and etiology of NIC in LMICs.

**Methods:**

We conducted a systematic literature review and meta-analyses of all eligible studies in 17 databases published from inception until April 2022 focusing on microbiologically confirmed NIC in LMICs.

**Findings:**

A total of 257 articles were included, with 10 994 NIC cases from 27 LMICs. The overall incidence rate was 2.6% (95% confidence interval [CI], 2.2–3.0). Regional disparities were evident, with South-East Asia reporting the highest incidence rate (6.3%; 95% CI, 3.2–10.3). The mean gestational age and birth weight were 31.4 weeks (standard deviation, 3.3) and 1530 g (standard deviation, 644.6), respectively. Among 10 087 included isolates, the predominant species was *C albicans* (39.0%), followed by *C parapsilosis* (24.8%), with marked differences in species distribution across World Health Organization regions. Fluconazole was the most commonly used agent for NIC treatment (55.4%; 1567/2826). Overall, 24.8% (1128/6613) of isolates with available data were resistant to fluconazole. The pooled estimated CFR was 18.7% (95% CI, 15.5–22.1).

**Conclusions:**

A higher NIC incidence rate and CFR in LMICs is noted compared to high-income countries, although infected babies were less premature with a higher birth weight. The proportion of fluconazole-resistant isolates was high. Prevention and treatment strategies for NIC need to be targeted to LMIC settings.

## Teaser

Our study highlights the higher incidence and case fatality rates of neonatal invasive candidiasis in low- and middle-income countries compared to high-income countries. Neonates were outside the well-defined high-risk group. Regional disparities in species and fluconazole resistances were observed.

Neonatal invasive candidiasis (NIC) is an important nosocomial infection associated with significant morbidity and mortality [1]. The incidence rate of NIC varies between 0.5% and 2% [2–5], with higher rates (7%–9%) in high-risk neonates (eg, gestational age <28 weeks or birth weight <1000 g) [3, 6, 7]. Most of the current data are derived from high-income countries (HICs). The burden of NIC in low- and middle-income countries (LMICs) remains poorly described [6]. Two recent studies, the Delhi Neonatal Infection Study and Global Neonatal Sepsis Observational Study, revealed different epidemiological characteristics of NIC in LMICs compared to HICs, with a higher incidence rate outside the high-risk group [8, 9]. Mortality rates can reach 40% for high-risk neonates [2, 10–12]. Despite the limited data on mortality associated with NIC from LMICs, this may be higher than in HICs [9, 13, 14].

The etiology of NIC in HICs is well described [15–17]. *Candida albicans* is the leading pathogen (40%–60% of all *Candida* species), followed by *Candida parapsilosis* (28%–42%). Fluconazole resistance for *C albicans* and *C parapsilosis* remains low (<5%) in HICs [18]. Globally, different epidemiology is observed with higher rates of non–*albicans Candida* isolates causing NIC in LMICs compared to HICs [19]. In addition, fluconazole-resistant NIC cases are increasingly being observed [20–22].

We aimed to address the critical knowledge gaps concerning NIC in LMICs, including burden, case-fatality rate (CFR), clinical and fungal epidemiology, as well as clinical management. Insights into these aspects could inform future policies and targeted research.

## METHODS

### Search Strategy

This systematic review and meta-analysis study was registered with PROSPERO (CRD42022318605). A quality assessment was undertaken using the Preferred Reporting Items for Systematic Reviews and Meta-Analyses guideline for reporting systematic reviews [23]. The databases searched included: Embase, PubMed, CENTRAL (Cochrane Central Register of Controlled Trials), Scopus, Web of Science, LILACS (Latin American and Caribbean Health Sciences Literature), WHOIRIS (World Health Organization [WHO] Library Dataset), Med-Carib, African Journals Online, African Index Medicus, Index Medicus for South-East Asia Region, Index Medicus for the Eastern Mediterranean Region, Western Pacific Region Index Medicus, OpenGrey, Google Scholar and WANFAN, and Airity library (for Chinese manuscripts). The search period ranged from each database's inception to 5 April 2022. The searches included the following concepts: “candidiasis”, “neonates”, “antifungal agents”, “fatality”, and “LMIC” with adjustments made as suitable to each database (detailed searches are listed in the Supplementary materials). Five reviewers (D.H.T.T., I.C.Y.W., B.D., L.F.C., and M.J.H.L.) independently screened titles and abstracts. The full-length articles were retrieved for final review and data extraction. Any disagreements were resolved with senior authors (L.F..A and Y.H.).

### Case Definition

To be more inclusive and capture the full spectrum of late-onset neonatal infections, we defined neonate as any infants aged up to 90 days. NIC was defined as a positive blood and/or cerebrospinal fluid (CSF) culture for *Candida* spp. Although antifungal susceptibility may vary, *Candida krusei*, *Candida glabrata,* and *Candida auris* were grouped as *Candida* spp. intrinsically resistant to fluconazole to assist analysis. The 2022 World Bank classification was used to define the country income level as LMICs; among these, further distinction into lower- and upper-middle-income countries was made [24].

### Selection Criteria

Original articles reporting NIC incidence, case fatality rate, and epidemiologic parameters were included. Eligible studies were retrospective studies, cohort studies, cross-sectional studies, and case series that reported 3 or more cases of NIC aged 0–90 days in LMICs. We excluded articles on nonneonatal populations, clinical trials, animal studies, case reports, and reviews. Studies were excluded if only aggregated data were presented. There was no language restriction. EndNote reference software (version X9, Philadelphia, PA; Clarivate) was used to manage articles.

### Data Extraction

Data collected included study design, publication year, study period, geographic location (WHO regions and countries) [25], total admitted neonates, total live births, total high-risk neonates, number of NIC cases, patient demographics, risk factors for NIC (prolonged hospital admission, known *Candida* colonization, parenteral nutrition, intravascular catheters, and prolonged use of broad-spectrum antibiotics), *Candida* species, susceptibility results, antifungal prophylaxis and treatment, length of hospital stay, and clinical outcomes. Preterm neonates were defined as neonates with gestational age of ≤37 weeks. High-risk neonates were defined as neonates with birth weight ≤1500 g and/or gestational age ≤28 weeks. Very low or extremely low birthweight (eLBW) was defined as ≤1500 or ≤1000 g at birth, respectively. Prolonged hospital admission was defined as a stay of more than 7 days. Prolonged use of broad-spectrum antibiotics was defined as neonates receiving carbapenem, third- or fourth-generation cephalosporin, or piperacillin-tazobactam for more than 5 days. The types of hospital (tertiary care hospital vs other facilities and public vs private centers) and the culture samples which were taken (blood vs cerebrospinal fluid) were also recorded. Data were extracted by one reviewer and verified by a second reviewer (D.H.T.T., I.C.Y.W., L.F.C., and M.J.H.L.).

### Risk of Bias Assessment

Risk of bias was assessed with the Risk of Bias In Non-Randomized Studies of Interventions Tool [26]. Two reviewers (D.H.T.T. and I.C.Y..W) independently completed the assessments for each reported outcome. Any conflicts were resolved by discussion with the senior authors (L.F.A. and Y.H.).

### Data Analysis

Descriptive analysis was performed to provide an epidemiological overview of NIC. Continuous variables were presented by means with standard deviation (SD), and categorical variables by numbers with percentages. We pooled the continuous variables and weighted them by their patient numbers. For those studies only reporting a median and the interquartile range (IQR), conversion was performed to estimate the sample mean and SD [27]. We used the number of neonates with candidemia or CSF-positive cultures (numerators) from all admitted neonates (denominators) to calculate incidence. We used the number of neonates with candidemia or CSF-positive cultures (denominators) and those cases who died (numerators) to estimate CFR. The meta-analysis with random-effects models was performed to estimate pooled incidences of NIC and CFR. The Freeman-Tukey double arcsine transformation was used to present regional and overall pooled estimates with 95% Wald confidence intervals (CI), heterogeneity using *I^2^* and test of significance of the overall pooled estimates [28–30]. All estimates were stratified by WHO regions and risk group. We defined all neonates as an overall neonatal population because some articles did not differentiate high-risk neonates from non–high-risk. Two sensitivity analysis were conducted, 1 included high-risk neonates and the other excluded Chinese studies because of the large amount of data from this country. Stata SE software (version 17. College Station, TX: StataCorp LLC) was used for data management and analyses.

## RESULTS

**Figure 1.**
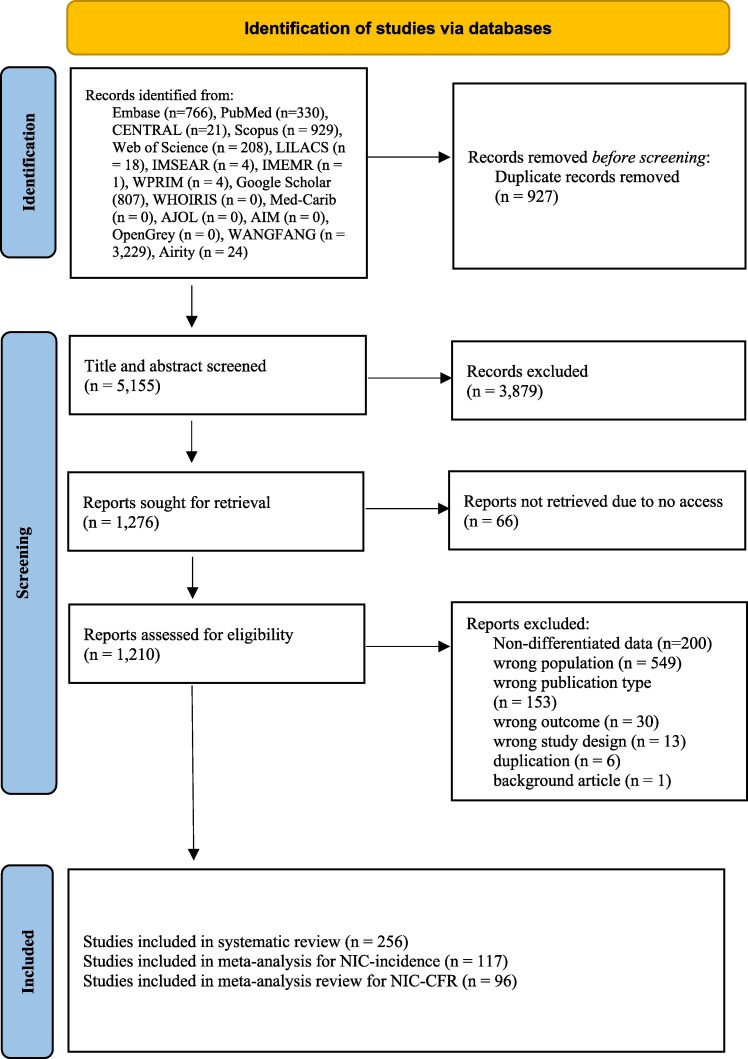
The PRISMA 2020 flow diagram for new systematic reviews that included searches of databases. Abbreviations: AIM, African Index Medicus; AJOL, African Journals Online; IMEMR, Index Medicus for the Eastern Mediterranean Region; IMSEAR, Index Medicus for the Eastern Mediterranean Region; LILACS, Latin American and Caribbean Health Sciences Literature; WHOIRIS, WHO Library Dataset; WPRIM, Western Pacific Region Index Medicus.

We assessed 1210 articles for eligibility, with 256 from 27 countries meeting the inclusion criteria for full-length review (Figure 1). Of those, 158 studies belonged to the Western Pacific WHO region, 31 from Latin America, 31 from Southeast Asia, 13 from Europe, 13 from the East Mediterranean, and 11 from the African region (Supplementary Tables 1 and 2). There were 117 studies reporting NIC incidence and 96 reporting CFR. Most studies reporting NIC incidence (75.2%; 88/117) and more than half reporting CFR (56.3%; 54/96) were conducted in China.

### Incidence of NIC and CFR

**Figure 2.**
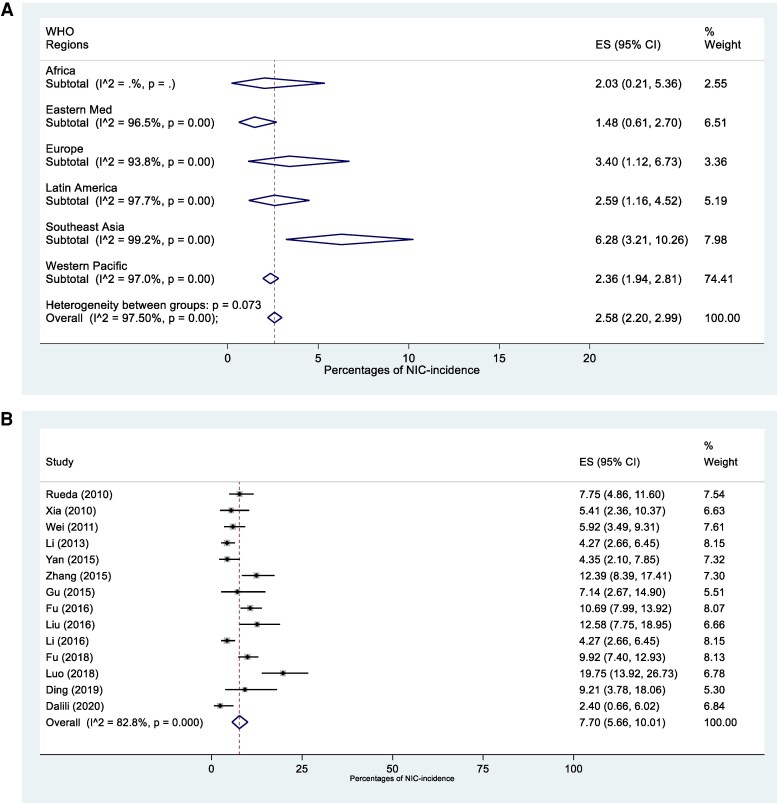
*A*, Pooled incidence of NIC in LMICs per WHO region. *B*, Pooled incidence of NIC in high-risk neonates in LMICs. Abbreviations: LMIC, lower and middle-income country; NIC, neonatal invasive candidiasis; WHO, World Health Organization.

The overall pooled estimated NIC incidence was 2.6% (95% CI, 2.2–3.0; *I*^2^, 97.5%). Similar rates were observed across regions except for South-East Asia with the highest reported incidence (6.3% [95% CI, 3.2–10.3], *I*^2^, 99.2%) (Figure 2*A*). The estimated incidence was higher in high-risk neonates (7.7% [95% CI, 5.7–10.0], *I*^2^, 82.8%) compared to the overall estimated incidence (Figure 2*B*).

**Figure 3.**
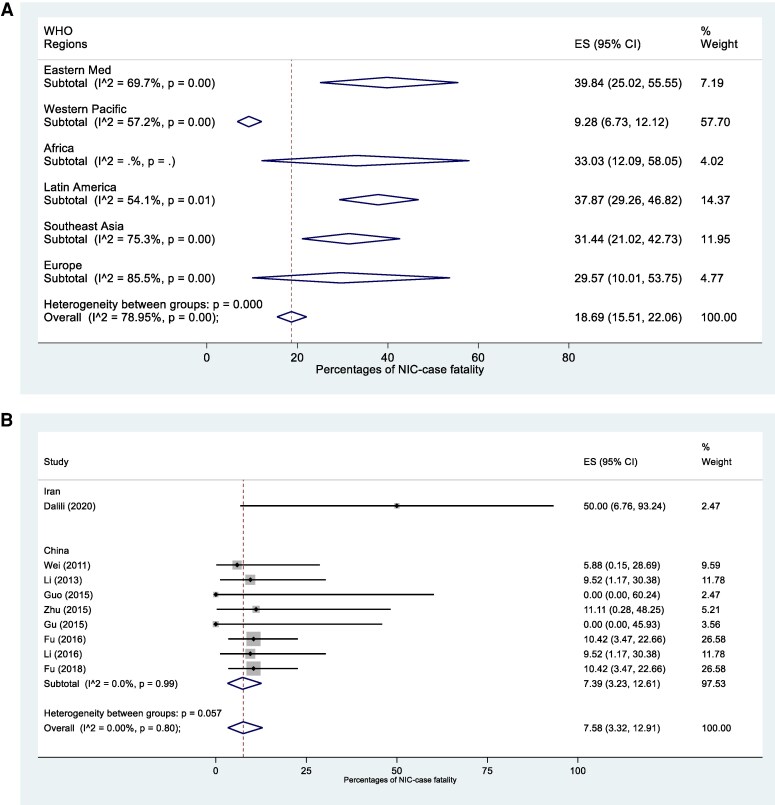
*A*, Pooled case-fatality rates (CFR) of NIC in LMICs per WHO region. *B*, Pooled case-fatality rates of NIC in high-risk neonates in LMICs. Abbreviations: LMIC, lower and middle-income country; NIC, neonatal invasive candidiasis; WHO, World Health Organization.

**Figure 4.**
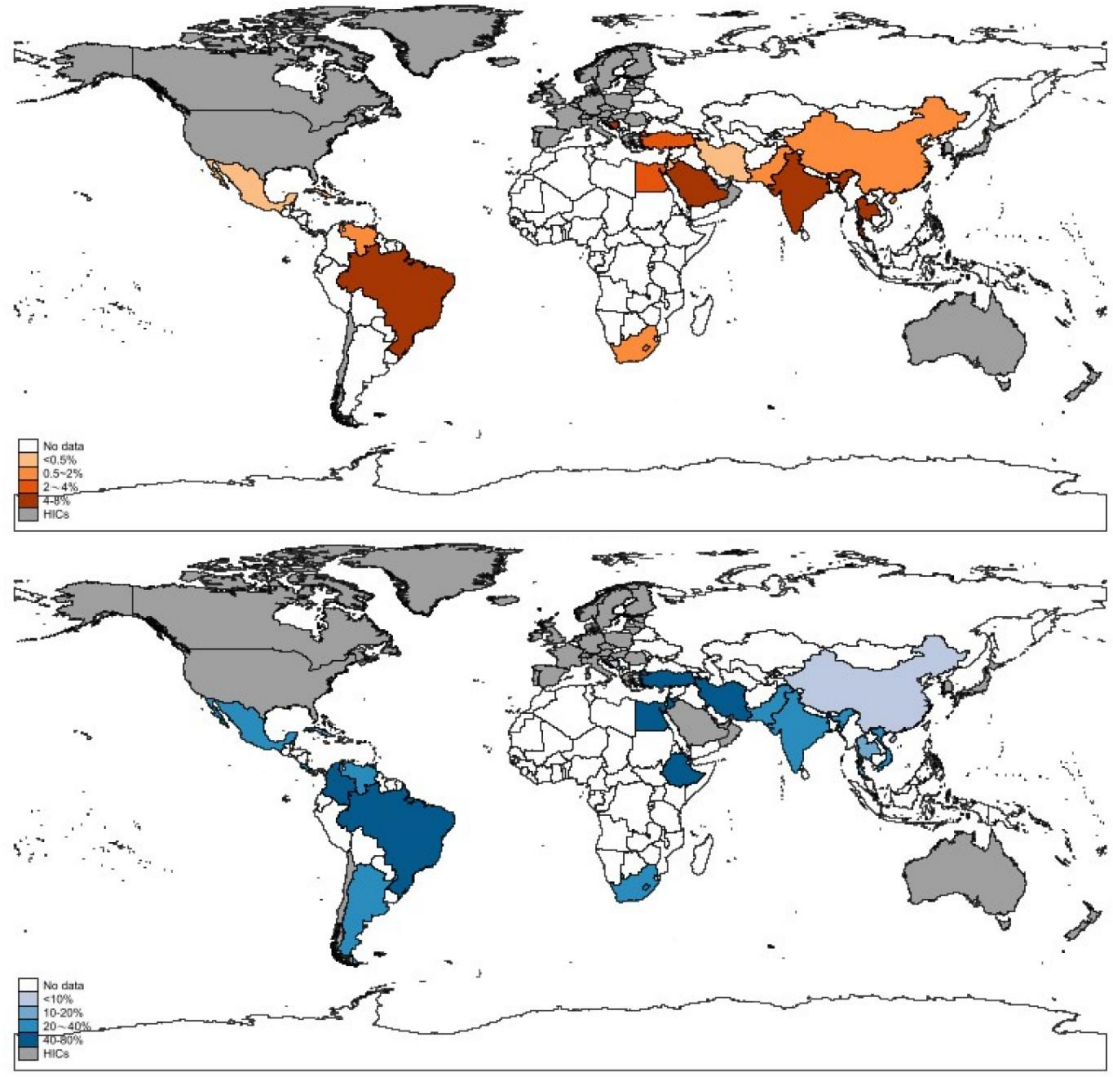
Incidence and CFR of NIC in LMICs by country in LMICs. Abbreviations: CFR, case-fatality rate; LMIC, lower and middle-income country; NIC, neonatal invasive candidiasis.

The overall estimated CFR was 18.7% (95% CI, 15.5–22.1; *I*^2^, 79.0%) (Figure 3*A*) with regional differences observed. The Eastern Mediterranean region had the highest reported CFR (39.8% [95% CI, 25.0–55.6], *I*^2^, 69.7%), followed by Latin America (37.9% [95% CI, 29.3–46.8], *I*^2^, 54.1%), Africa (33.0% [95% CI, 12.1–58.1]), South-East Asia (31.4% [95% CI, 21.0–42.7], *I*^2^, 75.2%), Europe (29.6% [95% CI, 10.0–53.8%], *I*^2^, 85.5%), and the Western Pacific region (9.3% [95% CI, 6.7–12.1], *I*^2^, 57.2%). For those studies with high-risk neonates data available, the estimated CFR was 7.6% (95% CI, 3.3–12.9; *I*^2^, 0%), although 8 of 9 studies were conducted in China (Figure 3*B*). Figure 4 presents the wide variation on NIC incidence and CFR among countries.

The subgroups analyses by low- and lower-middle-income countries versus upper-middle-income countries showed a pooled incidence twice as high in the former group (4.2% [95% CI, 2.5–6.4], *I*^2^, 98.9% vs 2.4% [95% CI, 2.0–2.8], *I*^2^, 97.1%). CFR was also higher in low and lower-middle-income countries, 37.5% (95% CI, 28.5-46.9; *I*^2^, 77.8%), compared to upper-middle-income countries, 15.0% (95% CI, 11.9-18.3; *I*^2^, 75.3%). Detailed data are presented in Supplementary Figures 1, 2, 3a, 3b, 4a, and 4b. The sensitivity analysis after excluding studies conducted in China showed a similar incidence of 3.3% (95% CI, 2.4–4.3; *I*^2^, 98.3%) (Supplementary Figure 5a). In contrast, the estimated CFR raised to 34.3% (95% CI, 29.0–39.9; *I*^2^, 78.6%) (Supplementary Figure 5b).

### Demographics and Clinical Characteristics

A total of 10 994 NIC cases were included. The majority were male (57.4%; 3268/5692). The mean age at diagnosis of NIC was 15.1 days of life (SD, 9.7); the mean gestational age was 31.4 weeks (SD, 3.3), and the mean birth weight was 1530.1 g (SD, 644.6). From those with data available, 66.8% (2530/3785) were preterm neonates, and only 12.7% (189/1488) were neonates with a gestational age of 28 weeks or less. Very low birth weight or eLBW was reported for 44.5% (1957/4402) of all the neonates with data available. Excluding the studies from China, the proportion of preterm neonates was 59.0% (1398/2369); the proportion of preterm neonates born ≤28 weeks of gestation and those with a birth weight ≤1500 g decreased to 7.5% (75/997) and 34.9% (1078/3093), respectively. A total of 11.8% (124/1054) cases had reported positive CSF cultures (with or without candidemia). All neonates with NIC and data available (5316/5316; 100%) were treated in tertiary hospitals, with 87.7% (3915/4466) admitted in high-dependency units and 79.1% (440/556) in public hospitals. The clinical characteristics and NIC risk factors are summarized in Table 1 (specific data on neonates with NIC from China are presented at Supplementary Table 3).

**Table 1.** Demographics and Clinical Characteristics for Neonatal Invasive Candidiasis

Patients’ Characteristics and Risk Factors	Overall Neonatal Population (n = 10 994)			Low- and Lower-middle Income Countries (n = 2341)			Upper-middle Income Countries (n = 8653)		
	No. of Countries/Regions^a^	No. of Neonates	Mean (SD)/n (%)	No. of Countries/Regions^a^	No. of Neonates	Mean (SD)/n (%)	No. of Countries/region^a^	No. of Neonates	Mean (SD)/n (%)
Patient characteristics									
Male	20	5692	3268 (57.4)	7	1576	976 (61.9)	13	4116	2294 (55.7)
Age, d	10	2568	15.1 (9.7)	2	177	14.6 (11.1)	8	2391	15.2 (9.6)
Gestational age (GA), wk	10	1789	31.4 (3.3)	2	296	31.5 (2.8)	8	1493	31.4 (3.4)
Birth weight, g	11	1627	1530.1 (644.6)	4	381	1508.1 (670.4)	7	1246	1536.9 (636.7)
Length of stay, d	4	2023	35.0 (21.9)	1	150	23.4 (10.3)	3	1873	35.9 (22.3)
vLBW or eLBW (<1500 g)	16	4402	1957 (44.5)	5	1146	264 (23.0)	11	3256	1693 (52.0)
Preterm neonates (GA < 37 wks)	20	3785	2530 (66.8)	6	1689	900 (53.3)	14	2096	1630 (77.8)
Extremely preterm neonates (GA < 28 wk)	10	1488	189 (12.7)	3	779	33 (4.2)	7	709	156 (22.0)
CSF *Candida* infection confirmed	7	1054	124 (11.8)	2	177	11 (4.2)	5	877	113 (12.9)
High dependency units	15	4466	3915 (87.7)	5	786	786 (100)	10	3660	3129 (85.5)
Tertiary care hospital	19	5316	5316 (100)	7	1625	1625 (100)	12	3691	3691 (100)
Public hospital	5	556	440 (79.1)	1	305	189 (61.7)	4	251	251 (100)
Risk factors for NIC									
Prolonged (≧7 d) hospital admission	9	717	499 (69.6)	3	127	74 (58.3)	6	590	425 (72.0)
Known *Candida* colonization	4	58	37 (63.8)	1	35	18 (51.4)	3	23	19 (82.6)
Receiving parenteral nutrition	16	3817	2283 (59.8)	4	553	203 (36.7)	12	3264	2080 (63.7)
Presence of a central venous catheter	17	4323	2457 (56.8)	5	724	313 (43.2)	12	2144	3599 (59.6)
Use of antibiotics	16	5472	3652 (66.7)	5	1599	950 (59.4)	11	3873	2702 (59.4)
Prolonged use of broad-spectrum antibiotic	10	1602	980 (61.2)	2	296	272 (91.9)	8	1306	708 (54.2)

Abbreviations: CSF, cerebrospinal fluid; eLBW, extremely low birth weight; GA, gestational age; NIC, neonatal invasive candidiasis; vLBW, very low birth weight.

For continuous variables such as age, gestational age, birth weight, and length of stay, we reported the mean (standard deviation). For categorical variables, we reported the count and percentage (n [%]).

^a^A study presented data from Argentina, Brazil, Chile, Colombia, Ecuador, Honduras, Mexico, and Venezuela.

### *Candida* Species Isolated

**Figure 5.**
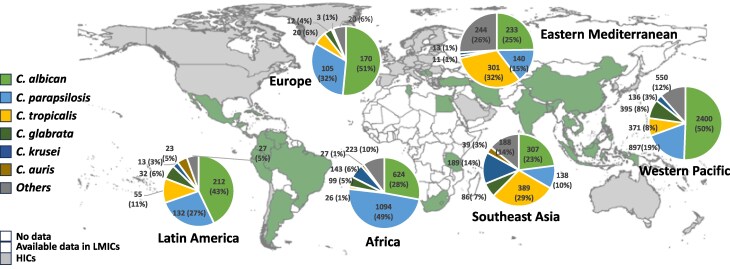
Distribution of the NIC isolates by WHO regions. Note: Numbers of studies included in each geography: Africa: 11; Latin America: 20; Eastern Mediterranean: 8; Europe: 7, Southeast Asia: 28; Western Pacific: 18. Abbreviations: NIC, neonatal invasive candidiasis; WHO, World Health Organization.

A total of 10 109 isolates were included. *C albicans* was the most common species (3946, 39.0%), followed by *C parapsilosis* (2506, 24.8%); *C tropicalis* (1162, 11.5%); *C glabrata* (renamed *Nakaseomyces glabratus*) (635, 6.3%); *C krusei* (renamed *Pichia kudriavzevii*) (497, 4.9%), and *C auris* (89, 0.9%). Regional differences in species distribution are illustrated in Figure 5. Other, less frequent *Candida* species (1274, 12.6%) are summarized in Supplementary Table 4.

A total of 56.5% (5715/10 109) isolates from 20 countries (20/27, 74.1%) had data on susceptibility to at least 1 antifungal agent. Overall, 24.8% (1128/4544) isolates were resistant to fluconazole, whereas 7.8% (165/2112), 6.1% (79/1285), and 2.3% (59/2566) showed resistance to voriconazole, itraconazole, and amphotericin B, respectively. The proportion with fluconazole resistance was highest in *C krusei* (203/281; 72.2%), followed by *C auris* (35/49; 71.4%), *C parapsilosis* (667/1616; 41.3%), *C glabrata* (37/279; 13.3%), *C tropicalis* (94/727; 12.9%) and *C albicans* (92/1592; 5.8%). Excluding *C krusei*, *C glabrata,* and *C auris*, the fluconazole resistance rate for other species was 21.7% (853/3935).

Marked regional variations in resistance were observed; South-East Asian region showing the highest proportion of fluconazole-resistant *Candida* spp isolates among those nonintrinsically resistant to fluconazole; 25.7% (48/187) of *C albicans,* 21.0% (21/100) of *C parapsilosis,* and 26.5% (83/313) of *C tropicalis* were fluconazole resistant. Notably, *C parapsilosis* had the highest fluconazole resistance in the African region, 57.8% (624/1079). Susceptibility testing results by WHO regions and *Candida* species are presented in Supplementary Table 5.

### Prevention and Management of NIC

The treatment of NIC was reported in 83 articles (2826 cases). The most common treatment prescribed was fluconazole (55.4%, 1567), followed by amphotericin B (27.1%, 767) and a combination of fluconazole-amphotericin B (13.4%, 378). Echinocandins were rarely prescribed (0.7%, 21). Detailed antifungal use by region is presented in Supplementary table 6.

Antifungal prophylaxis was reported in 25 articles, from 7 countries, of which 404 NIC cases (22.8%; 404/1774) received antifungal prophylaxis. From these 25 studies, 17 were conducted in China. A higher proportion of high-risk neonates received prophylaxis (85/107, 79.4%). Three of 4 studies reporting antifungal prophylactic use in high-risk neonates were conducted in China. In most cases (399/404, 98.8%), fluconazole was the agent of choice. Antifungal prophylaxis in NIC cases was reported less frequently in low or lower-middle-income countries (54/1,008, 5.4%) compared to upper-middle-income countries (345/756, 45.6%).

## DISCUSSION

This systematic review assessed the disease burden, clinical characteristics, and outcomes of NIC in LMICs. Data from 27 countries and 10 994 cases showed that the overall incidence was 2.6%, with marked regional differences. The overall CFR was 18.7%, ranging from 9% to 40% across WHO regions. Where gestational age was reported, the majority of cases occurred in neonates who are not traditionally categorized as high risk for NIC; with only 12.7% of neonates born before 28 weeks and a mean gestational age and birth weight of 31.4 weeks and 1530.1 g, respectively. Overall, *C albicans* was the most common isolate, but non-*albicans Candida* species were more prevalent in some WHO regions. Fluconazole-resistant isolates account for a quarter of all the isolates and for more than one fifth of the isolates excluding *C krusei*, *C glabrata,* and *C auris*. Whereas fluconazole-resistant *C albicans* was reported in only a small proportion of the cases (5.8%), fluconazole resistance for *C parapsilosis* was as high as 41.3%. South-East Asian and African regions showed the highest fluconazole resistance rates with variability among the species. The vast majority (95.1%) of the neonates received fluconazole and amphotericin B for treatment, whereas echinocandins were rarely used.

Our results have shown a higher incidence rate of NIC in LMICs (approximately 3%) among admitted neonates compared to published data from HICs (0.5%–2%) [2–4]. The South-East Asia region has the highest reported incidence rate, 6%. Several possible reasons could explain the difference in NIC burden between LMICs and HICs. First of all, fewer resources available to implement targeted antifungal prophylaxis and other strategies to prevent healthcare-associated infections [2, 6, 19]. These interventions have played a crucial role in the reduction of NIC incidence in HICs during the past 2 decades [2, 31–33]. Second, the colonization pressure differs; in HICs, *Candida* spp. colonization occurs in ∼26.7%–62.5% of critically ill neonates within the first 2 weeks of life. In contrast, data from India or South Africa have shown higher and earlier rates of colonization [34, 35]. Finally, the role of *Candida* vertical transmission, although not fully well-described, some reports suggest its potential contribution to the incidence of NIC, including the transmission of resistant isolates [36]. Previous studies of NIC in HICs have estimated a CFR of approximately 20% [17]. Our sensitivity analysis, once the studies conducted in China were removed, has shown a higher CFR in LMICs, up to 40%. The differences might be multifactorial; gaps in recognition, diagnosis and management of NIC, differences in neonatal care-seeking behaviors or general neonatal health care interventions, and weak infrastructures and health systems [37, 38]. This emphasizes the need to tailor interventions on NIC in resource-limited settings.

The low gestational age and birth weight are the main risk determinants of NIC [2, 7]. However, the Delhi Neonatal Infection Study reported high rates of NIC for outborn neonates, of whom most were older than 32 weeks’ gestation (73.3%) or had a birth weight over 1500 g (61.5%) [8]. Furthermore, a multicountry study, the Global Neonatal Sepsis Observational Study [9], showed that in 127 neonates with NIC, the median gestational age at birth was 30 weeks (IQR: 28–34) and the median birthweight was 1270 g (IQR: 990– 1692); only 27.0% of all neonates had a birthweight below 1000 g [9]. Our findings have shown similar epidemiological characteristics of NIC in LMICs; with only 12.7% of all NIC cases born at less than 28 weeks, the mean gestational age of 31.4 (SD, 3.3) weeks and the mean birthweight of 1524.2 (SD, 644.0). This contrasts with other large cohorts from HICs, such as EUROCANDY [15], where the median age was 27 weeks (IQR, 10). The most plausible explanation might be that more premature and eLBW neonates do not survive long enough to develop NIC [38]. Other factors playing into this are limitations on neonatal infection prevention and care bundles, especially primary prevention interventions, which include the care of central line catheters or other medical devices, as well as the reduction in the use of broad-spectrum antibiotics and adequate handling [39].

Globally, the majority of the cases of candidemia are now attributed to 6 main species, *C albicans, C glabrata, C tropicalis, C parapsilosis, C auris,* and *C krusei*. However, population-based studies have demonstrated that the distribution of these species varies between geographical regions [40]. In children and neonates, whereas *C albicans* remains the most prevalent species, *C parapsilosis*, *C tropicalis* [15, 41–43] and, in certain settings, *C auris* are becoming more common [9, 22]. Despite the lack of temporal trends and exhibiting regional variations, our findings have demonstrated that non-*albicans* species were more prevalent than *C albicans* in LMICs. About 25% of all isolates were resistant to fluconazole. The South-East Asian region had the highest rates of fluconazole-resistant isolates. The emergence of these species and resistant isolates is likely multifactorial; complex health care systems, global warming, behavioral factors with extensive use of fluconazole in public health settings or unregulated sale and use of antifungals, different spectrum of comorbidities in certain regions, inadequate infection prevention practices, and prolonged use and overuse of antifungals across the One Health spectrum, especially azoles [22, 44–47]. These global changes in *Candida* species epidemiology have clear clinical implications, particularly in LMICs, where the availability of antifungal medicines may be limited and fluconazole remains the principal agent for targeted prophylaxis and treatment of NIC [44, 48–52].

Data from the Global Antimicrobial Resistance, Prescribing, and Efficacy in Neonates and Children—Point Prevalence Survey showed that the use of fluconazole was higher in LMICs compared with HICs (66.8% vs 39.2% of all prescriptions), whereas other triazoles, amphotericin B, and echinocandins were more commonly used in HIC [53]. Our findings confirm that fluconazole (55.4%) and amphotericin B (27.1%) are the most used treatment modalities. The use of echinocandins was reported in less than 1% of the cases, despite their extended-spectrum against *Candida* spp. including *C auris*, efficacy against *Candida* biofilms and a favorable safety profile [6, 54].

There are limitations to our review. First, the robustness of our results relies on the availability and quality of the included studies. There is likely selection and reporting bias. Selection bias, because many countries did not provide data, and most of the studies were from tertiary-level hospitals. Reporting bias, because data are likely affected by a degree of underestimation of the “true” incidence of disease. We encountered significant heterogeneity. Moreover, there was also variation in the definitions of the neonatal population, in the calculation of gestational age and in the reporting and methodologies employed to identify the species or define isolate susceptibility. A few key clinical variables were incompletely reported in this review. For example, gestational age was available for only 1789 of 10 994 case (16.3%), birthweight data were available for 1627 cases (14.8%). Similarly, information on specific risk factors such as *Candida* colonization, was reported in only a small subset of cases.

However, we have conducted several sensitivity analyses to reduce this heterogeneity. Furthermore, we were unable to determine any temporal trends of particular significance when assessing the emergence of fluconazole-resistant isolates. Moreover, *Candida* species have the potential to drive infection outbreaks, although we did a sensitivity analysis after identifying the manuscripts where the word “outbreak” was mentioned. Only 9 papers included some sort of outbreak data. None of the papers reporting outbreak data had been included in the incidence analysis. A total of 8 papers including outbreak data were excluded for CRF calculation, with a lower CRF of 17.8% (95% CI, 14.7–21.1). Finally, a significant number of included studies were conducted in China. A sensitivity analysis was performed to avoid publication bias. After excluding studies conducted in China, there were a similar incidence of 3.3% but a raised CFR of 34.3%. Whereas China is classified as an LMIC [24], there are differences in healthcare services between China and other countries in the Global South [55].

In 2022, the WHO published the Fungal Priority Pathogens List to systematically prioritize fungal pathogens, considering their unmet needs in research and development and perceived global public health importance [44, 56]. Critical and high-priority *Candida* species (eg, *C albicans*, *C auris*, *C parapsilosis*) affect neonatal health globally, but disproportionately in LMICs [44, 51, 57–59]. Nevertheless, there is a paucity of epidemiological data, clinical phase III trials hardly include neonates, delay in access to newly developed antifungal medicines, and access to antifungal therapy is severely limited in LMIC [50, 56]. From our data and building on the previous evidence, several specific recommendations are proposed: (1) the integration of NIC research into broader platform studies focused on neonatal sepsis and antimicrobial resistance in neonates, as this will allow a more efficient approach compared to traditional siloed studies. (2) The design of studies to assess the colonization pressure for *Candida* species in neonatal units in resource-limited settings. (3) Prospective epidemiological data collection to define the at-risk population for NIC in LMICs, facilitating a risk-based approach for future interventional studies. (4) The study and subsequent implementation of targeted infection prevention and care bundles for NIC in neonates in LMICs. This should include simple, low-cost, and evidence-based interventions such as breastfeeding, kangaroo mother-child care or the use of probiotics, considering the rising fluconazole resistance rates and limited access to alternative agents. (5) The better understanding on the long-term prognosis of children affected with NIC in LMIC.

In conclusion, we emphasize the importance of NIC as a significant contributor to neonatal morbidity and mortality in LMICs, where its true burden is likely underestimated. Collaborative efforts and increased research investment are imperative to identify high-risk neonates in resource-limited settings and implement targeted preventive measures and optimal management strategies.

## Supplementary Material

ofaf329_Supplementary_Data

## Supplementary Data

Supplementary materials are available at *Open Forum Infectious Diseases* online. Consisting of data provided by the authors to benefit the reader, the posted materials are not copyedited and are the sole responsibility of the authors, so questions or comments should be addressed to the corresponding author.

## Notes

***Acknowledgments*.** Julia Bielicki (Centre for Neonatal and Paediatric Infection, St George's, University of London, UK, and Paediatric Research Centre, University of Basel Children's Hospital, Basel, Switzerland); Louise F. Hill (Centre for Neonatal and Paediatric Infection, City St George's, University of London, UK).

***Patient consent.*** This is a review and meta-analysis piece; it does not include factors necessitating patient consent.

***Financial support.*** This work was supported by ESPID (European Society of Paediatric Infectious Diseases) Systematic Review Grant from ESPID, 2021.

## References

[1] Greenberg R, Benjamin DK Jr. Neonatal candidiasis: diagnosis, prevention and treatment. J Infect 2014; 69(Suppl 1):S19–22. 25129318 10.1016/j.jinf.2014.07.012 PMC4252884

[2] Aliaga S, Clark RH, Laughon M, et al Changes in the incidence of candidiasis in neonatal intensive care units. Pediatrics 2014; 133:236–42. 24446441 10.1542/peds.2013-0671 PMC3904270

[3] Barton M, O’Brien K, Robinson JL, et al Invasive candidiasis in low birth weight preterm infants: risk factors, clinical course and outcome in a prospective multicenter study of cases and their matched controls. BMC Infect Dis 2014; 14:1–10. 24924877 10.1186/1471-2334-14-327 PMC4063435

[4] Kelly MS, Benjamin DK, Smith PB. The epidemiology and diagnosis of invasive candidiasis among premature infants. Clin Perinatol 2015; 42:105–17. 25677999 10.1016/j.clp.2014.10.008 PMC4328135

[5] Cotten CM, McDonald S, Stoll B, Goldberg RN, Poole K, Benjamin DK Jr. The association of third-generation cephalosporin use and invasive candidiasis in extremely low birth-weight infants. Pediatrics 2006; 118:717–22. 16882828 10.1542/peds.2005-2677

[6] Kilpatrick R, Scarrow E, Hornik C, Greenberg RG. Neonatal invasive candidiasis: updates on clinical management and prevention. Lancet Child Adolesc Heal 2022; 6:60–70. 10.1016/S2352-4642(21)00272-8 34672994

[7] Benjamin DK, Stoll BJ, Gantz MG, et al Neonatal candidiasis: epidemiology, risk factors, and clinical judgment. Pediatrics 2010; 126:1–18. 20876174 10.1542/peds.2009-3412 PMC3045840

[8] Jajoo M, Manchanda V, Chaurasia S, et al Alarming rates of antimicrobial resistance and fungal sepsis in outborn neonates in North India. PLoS One 2018; 13:1–16. 10.1371/journal.pone.0180705 PMC6023165 29953451

[9] Cook A, Ferreras-Antolin L, Adhisivam B, et al Neonatal invasive candidiasis in low- and middle-income countries: data from the {NeoOBS} study. Med Mycol 2023 Mar; 61:311–22. 10.1093/mmy/myad010 PMC10026246 36881725

[10] Autmizguine J, Smith PB, Prather K, et al Effect of fluconazole prophylaxis on fluconazole Candida susceptibility in premature infants. J Antimicrob Chemother 2018; 73:1–6. 30247579 10.1093/jac/dky353 PMC6927883

[11] Adams-Chapman I, Bann CM, Das A, et al Neurodevelopmental outcome of extremely low birth weight infants with Candida infection. J Pediatr 2013; 163:961–7. 23726546 10.1016/j.jpeds.2013.04.034 PMC3786056

[12] Stoll BJ, Hansen N, Fanaroff AA, et al Late-onset sepsis in very low birth weight neonates: the experience of the NICHD Neonatal Research Network. Pediatrics 2002; 110(2 I):285–91. 12165580 10.1542/peds.110.2.285

[13] Ahangarkani F, Shokohi T, Rezai MS, et al Epidemiological features of nosocomial candidaemia in neonates, infants and children: a multicentre study in Iran. Mycoses 2020; 63:382–94. 31985076 10.1111/myc.13053

[14] Ballot DE, Bosman N, Nana T, Ramdin T, Cooper PA. Background changing patterns of neonatal fungal sepsis in a developing country. J Trop Pediatr 2013; 59:6–10. 10.1093/tropej/fmt053 23803724

[15] Warris A, Pana Z-D, Oletto A, et al Etiology and outcome of candidemia in neonates and children in Europe: an 11-year multinational retrospective study. Pediatr Infect Dis J 2020; 39:114–20. 31725552 10.1097/INF.0000000000002530 PMC7208278

[16] Steinbach WJ, Roilides E, Berman D, et al Results from a prospective, international, epidemiologic study of invasive candidiasis in children and neonates. Pediatr Infect Dis J 2012; 31:1252–7. 22982980 10.1097/INF.0b013e3182737427

[17] Pana ZD, Roilides E, Warris A, Groll AH, Zaoutis T. Epidemiology of invasive fungal disease in children. J Pediatric Infect Dis Soc 2017; 6:S3–11. 28927200 10.1093/jpids/pix046 PMC5907880

[18] Warris A, Pana ZD, Oletto A, Lundin R. Antifungal drug susceptibility of candida spp. In neonatal and paediatric candidaemia: a european multi-centre retrospective study (EUROCANDY). Presented at: ESPID Annual Conference. 2018. p. 97.

[19] Kaur H, Chakrabarti A. Strategies to reduce mortality in adult and neonatal candidemia in developing countries. J Fungi 2017; 3:43. 10.3390/jof3030041 PMC5715942 29371558

[20] Chakrabarti A, Singh S. Multidrug-resistant *Candida auris*: an epidemiological review. Expert Rev Anti Infect Ther 2020; 18:551–62. 32237924 10.1080/14787210.2020.1750368

[21] Govender NP, Patel J, Magobo RE, et al Emergence of azole-resistant *Candida parapsilosis* causing bloodstream infection: results from laboratory-based sentinel surveillance in South Africa. J Antimicrob Chemother 2016; 71:1994–2004. 27125552 10.1093/jac/dkw091 PMC11911919

[22] Van Schalkwyk E, Mpembe RS, Thomas J, et al Epidemiologic shift in candidemia driven by *Candida auris*, South Africa, 2016–2017. Emerg Infect Dis 2019; 25:1698–707. 31441749 10.3201/eid2509.190040 PMC6711229

[23] Page MJ, McKenzie JE, Bossuyt PM, et al The PRISMA 2020 statement: an updated guideline for reporting systematic reviews. BMJ 2021; 372:n71. 33782057 10.1136/bmj.n71 PMC8005924

[24] World Bank Country and Lending Groups [Internet]. World Bank Data Help Desk. 2022. Available at: https://datahelpdesk.worldbank.org/knowledgebase/articles/906519-world-bank-country-and-lending-groups

[25] World Health Organization - Countries overview [Internet]. World Health Organization. Available at: https://www.who.int/countries

[26] Sterne JA, Hernán MA, Reeves BC, et al ROBINS-I: a tool for assessing risk of bias in non-randomised studies of interventions. BMJ 2016; 355:4–10. 10.1136/bmj.i4919 PMC5062054 27733354

[27] Wan X, Wang W, Liu J, Tong T. Estimating the sample mean and standard deviation from the sample size, median, range and/or interquartile range. BMC Med Res Methodol 2014; 14:135. 25524443 10.1186/1471-2288-14-135 PMC4383202

[28] Zhou Q, Li Q, Meng W, Luo Z, Chen Y. Statistical concerns for meta-analysis of rare events and small sample sizes. Lancet Infect Dis 2022; 22:1111–2. 10.1016/S1473-3099(22)00363-2 35870454

[29] Nyaga VN, Arbyn M, Aerts M. Metaprop: a Stata command to perform meta-analysis of binomial data. Arch Public Heal 2014; 72:1–10. 10.1186/2049-3258-72-39 PMC4373114 25810908

[30] Sherwood E, Vergnano S, Kakuchi I, et al Invasive group A streptococcal disease in pregnant women and young children: a systematic review and meta-analysis. Lancet Infect Dis 2022; 22:1076–88. 35390294 10.1016/S1473-3099(21)00672-1 PMC9217756

[31] Benedict K, Jackson BR, Chiller T, Beer KD. Estimation of direct healthcare costs of fungal diseases in the United States. Clin Infect Dis 2019; 68:1791–7. 30204844 10.1093/cid/ciy776 PMC6409199

[32] Oeser C, Lamagni T, Heath PT, Sharland M. The epidemiology of neonatal and pediatric candidemia. Pediatr Infect Dis J 2013; 32:32–5. 23241987 10.1097/INF.0b013e318275612e

[33] Ting JY, Roberts A, Synnes A, et al Invasive fungal infections in neonates in Canada: epidemiology and outcomes. Pediatr Infect Dis J 2018; 37:1154–9. 29561508 10.1097/INF.0000000000001968

[34] Singh K, Chakrabarti A, Narang A, Gopalan S. Yeast colonisation and fungaemia in preterm neonates in a tertiary care centre. Indian J Med Res 1999; 110:169–73. 10680302

[35] Mabena FC, Olwagen CP, Phosa M, et al Bacterial and Candida colonization of neonates in a regional hospital in South Africa. Pediatr Infect Dis J 2023; 43:e85–8. 10.1097/INF.0000000000004177 38381956

[36] Azevedo MJ, Araujo R, Campos J, et al Vertical transmission and antifungal susceptibility profile of yeast isolates from the oral cavity, gut, and breastmilk of mother–child pairs in early life. Int J Mol Sci 2023; 24:1–13. 10.3390/ijms24021449 PMC9867488 36674962

[37] Herbert HK, Lee AC, Chandran A, Rudan I, Baqui AH. Care seeking for neonatal illness in low- and middle-income countries: a systematic review. PLoS Med 2012; 9:e1001181. 22412355 10.1371/journal.pmed.1001183 PMC3295826

[38] Sharrow D, Hug L, You D, et al Global, regional, and national trends in under-5 mortality between 1990 and 2019 with scenario-based projections until 2030: a systematic analysis by the UN Inter-agency Group for Child Mortality Estimation. Lancet Glob Heal 2022; 10:e195–206. 10.1016/S2214-109X(21)00515-5 PMC8789561 35063111

[39] Molina García A, Cross JH, Fitchett EJA, et al Infection prevention and care bundles addressing health care-associated infections in neonatal care in low-middle income countries: a scoping review. eClinicalMedicine 2022; 44:101259. 35059614 10.1016/j.eclinm.2021.101259 PMC8760419

[40] Guinea J . Global trends in the distribution of Candida species causing candidemia. Clin Microbiol Infect 2014; 20:5–10. 10.1111/1469-0691.12539 24506442

[41] Blyth C, Hale K, Palasanthiran P, Brien OT, Mh B. Antifungal therapy in infants and children with proven, probable or suspected invasive fungal infections (review). Cochrane Database Syst Rev 2010; 2010:CD006343. 20166083 10.1002/14651858.CD006343.pub2 PMC10576261

[42] Santolaya ME, Alvarado T, Queiroz-Telles F, et al Active surveillance of candidemia in children from Latin America: a key requirement for improving disease outcome. Pediatr Infect Dis J 2014; 33:40–4. 10.1097/INF.0000000000000039 23995591

[43] Wattier RL, Dvorak CC, Hoffman JA, et al A prospective, international cohort study of invasive mold infections in children. J Pediatric Infect Dis Soc 2015; 4:313–22. 26582870 10.1093/jpids/piu074 PMC4681382

[44] World Health Organization. WHO fungal priority pathogens list to guide research, development and public health action [Internet]. 2022. Available at: https://www.who.int/publications/i/item/9789240060241

[45] Castelo-Branco D, Lockhart SR, Chen Y-C, et al Collateral consequences of agricultural fungicides on pathogenic yeasts: a One Health perspective to tackle azole resistance. Mycoses 2022; 65:303–11. 34821412 10.1111/myc.13404 PMC11268486

[46] Casadevall A, Kontoyiannis DP, Robert V. Collateral consequences of agricultural fungicides on pathogenic yeasts: a One Health perspective to tackle azole resistance. MBio 2019; 10:1–7. 10.1111/myc.13404 PMC11268486 34821412

[47] Verma R, Pradhan D, Hasan Z, Singh H, Jain AK, Khan LA. A systematic review on distribution and antifungal resistance pattern of *Candida* species in the Indian population. Med Mycol 2021; 59:1145–65. 34625811 10.1093/mmy/myab058

[48] Fisher MC, Alastruey-Izquierdo A, Berman J, et al Tackling the emerging threat of antifungal resistance to human health. Nat Rev Microbiol 2022; 20:557–71. 35352028 10.1038/s41579-022-00720-1 PMC8962932

[49] Pfaller MA, Diekema DJ, Turnidge JD, Castanheira M, Jones RN. Twenty years of the SENTRY antifungal surveillance program: results for Candida species from 1997–2016. Open Forum Infect Dis 2019; 6(Suppl 1):S79–94. 30895218 10.1093/ofid/ofy358 PMC6419901

[50] Kneale M, Bartholomew JS, Davies E, Denning DW. Global access to antifungal therapy and its variable cost. J Antimicrob Chemother 2016; 71:3599–606. 27516477 10.1093/jac/dkw325

[51] Daneshnia F, de Almeida Júnior JN, Ilkit M, et al Worldwide emergence of fluconazole-resistant *Candida parapsilosis*: current framework and future research roadmap. Lancet Microbe 2023; 4:e470–80. 37121240 10.1016/S2666-5247(23)00067-8 PMC10634418

[52] Hope WW, Castagnola E, Groll AH, et al ESCMID guideline for the diagnosis and management of Candida diseases 2012 : prevention and management of invasive infections in neonates and children caused by Candida spp. Clin Microbiol Infect 2012; 18:38–52. 23137136 10.1111/1469-0691.12040

[53] Ferreras-Antolin L, Bielicki J, Warris A, Sharland M, Hsia Y. Global divergence of antifungal prescribing patterns: data from the global antimicrobial resistance, prescribing, and efficacy in neonates and children surveys. Pediatr Infect Dis J 2021; 40:327–32. 33710977 10.1097/INF.0000000000002983

[54] Botero-Calderon L, Benjamin D, Coher-Wolkowiez M. Advances in the treatment of invasive neonatal candidiasis. Expert Opin Pharmacother 2015; 16:1035–48. 25842986 10.1517/14656566.2015.1031108 PMC4402277

[55] Yip W, Fu H, Jian W, et al Universal health coverage in China part 1: progress and gaps. Lancet Public Heal 2023; 8:e1025–34. 10.1016/S2468-2667(23)00254-2 38000882

[56] World Health Organization. GLASS-FUNGI Module. 2022. Available at: https://www.who.int/initiatives/glass/glass-modules-5

[57] Pappas PG, Kauffman CA, Andes DR, et al Clinical practice guideline for the management of candidiasis : 2016 update by the Infectious Diseases Society of America. Clin Infect Dis 2016; 62:1–50. 26679628 10.1093/cid/civ933 PMC4725385

[58] Lockhart SR, Etienne KA, Vallabhaneni S, et al Simultaneous emergence of multidrug-resistant *Candida auris* on 3 continents confirmed by whole-genome sequencing and epidemiological analyses. Clin Infect Dis 2017; 64:134–40. 27988485 10.1093/cid/ciw691 PMC5215215

[59] Forgács L, Borman AM, Kovács R, et al In vivo efficacy of amphotericin B against four *Candida auris* clades. J Fungi 2022; 8:499. 10.3390/jof8050499 PMC9144575 35628754

