# Supplementary material for: The Burden of Neonatal Invasive Candidiasis in Low- and Middle-income Countries: A Systematic Review and Meta-analysis
Source: Open Forum Infect Dis. 2025 Jun 11;12(7):ofaf329. doi: 10.1093/ofid/ofaf329 (PMC12236162; doi:10.1093/ofid/ofaf329)
Supplement: ofaf329_Supplementary_Data [file ofaf329_supplementary_data.docx]

**Supplementary materials**

Supplementary Figure 1: Pooled incidence of NIC in LMICs.

Note: data was from 14 countries

Supplementary Figure 2: Pooled case-fatality rates (CFR) of NIC in LMICs.

Note: data was from 14 countries

Supplementary Figure 3a. Sensitivity analysis of pooled incidence of NIC in upper-middle income countries

Supplementary Figure 3b. Sensitivity analysis of pooled incidence of NIC in low- and lower-middle income countries

Supplementary Figure 4a. Sensitivity analysis of pooled CFR of NIC in upper-middle income countries

Supplementary Figure 4b Sensitivity analysis of pooled CFR of NIC in low- and lower-middle income countries

Supplementary Figure 5a Sensitivity analysis of pooled incidence of NIC in LMICs, excluding studies conducted in China.

Supplementary Figure 5b Sensitivity analysis of pooled CFR of NIC in LMICs, excluding studies conducted in China.

Table S1 The number of studies (and number of neonates) by country in the incidence analysis

| Countries | Number of studies | Number of neonates | Number of cases |
| --- | --- | --- | --- |
| Bosnia and Herzegovina | 1 | 921 | 48 |
| Brazil | 3 | 1,910 | 83 |
| China | 88 | 214,309 | 2206 |
| Cuba | 1 | 7,219 | 15 |
| Egypt | 3 | 1,733 | 49 |
| India | 7 | 43,453 | 474 |
| Iran | 2 | 3,515 | 6 |
| Jordan | 1 | 8,862 | 24 |
| Mexico | 1 | 37,462 | 85 |
| Pakistan | 2 | 6,379 | 86 |
| South Africa | 3 | 1,921 | 23 |
| Thailand | 1 | 229 | 13 |
| Turkey | 3 | 3,165 | 55 |
| Venezuela | 1 | 128 | 2 |

Table S2 The number of studies (and number of neonates) by country in the CFR analysis

| Countries | Number of studies | Number of neonates | Number of death cases |
| --- | --- | --- | --- |
| Argentina | 1 | 19 | 5 |
| Bosnia and Herzegovina | 1 | 48 | 4 |
| Brazil | 7 | 132 | 60 |
| China | 54 | 1,646 | 207 |
| Colombia | 2 | 11 | 6 |
| Costa Rica | 2 | 119 | 38 |
| Cuba | 1 | 15 | 2 |
| Egypt | 2 | 48 | 22 |
| Ethiopia | 1 | 17 | 13 |
| India | 9 | 541 | 166 |
| Iran | 4 | 51 | 23 |
| Jordan | 1 | 24 | 13 |
| Mexico | 1 | 85 | 28 |
| Pakistan | 1 | 45 | 11 |
| South Africa | 2 | 1,526 | 330 |
| Thailand | 2 | 25 | 3 |
| Turkey | 3 | 72 | 29 |
| Venezuela | 1 | 12 | 4 |
| Vietnam | 1 | 90 | 24 |

Table S3. Demographics and clinical characteristics for neonatal invasive candidiasis in China

| Patients’ characteristics and risk factors | Overall neonatal population (n=3,888) | |
| --- | --- | --- |
|  | No. of Neonates | Mean (SD)/  n (%) |
| Patient characteristics | | |
| Male sex | 1,968 | 1,174 (59.7) |
| Age, days | 811 | 16.2 (11.2) |
| Gestational age (GA), weeks | 1,187 | 31.2 (3.1) |
| Birth weight, grams | 1,006 | 1,525.9 (594.0) |
| Length of stay, days | 326 | 46.7 (28.3) |
| vLBW or eLBW (<1,500g) | 1,310 | 879 (67.1) |
| Preterm neonates (GA< 37 weeks) | 1,416 | 1,132 (79.9) |
| Extremely preterm neonates  (GA < 28 weeks) | 491 | 114 (23.2) |
| CSF infection | 737 | 124 (11.8) |
| High dependency units | 1,658 | 3,915 (87.7) |
| Tertiary care hospital | 1,285 | 5,316 (100) |
| Public hospital | NA | 440 (79.1) |
| Risk factors for NIC | | |
| Prolonged hospital admission | 445 | 287 (64.5) |
| Known colonisation | 9 | 0 |
| Receiving parenteral nutrition | 1,202 | 990 (82.4) |
| Presence of a central catheter | 1,488 | 1,071 (72.0) |
| Use of antibiotics | 1,793 | 1,367 (76.2) |
| Prolong use of broad-spectrum antibiotic | 1,041 | 530 (50.9) |

Note: GA; gestational age, vLBW; very low birth weight eLBW; extremely low birth weight, CSF; cerebrospinal fluid, NIC; neonatal invasive candidiasis

Table S4 The distribution of other NIC species by WHO regions.

|  | Africa | Latin America | Eastern Mediterranean | Europe | South-East Asia | Western Pacific | Total isolates |
| --- | --- | --- | --- | --- | --- | --- | --- |
| ***C. pelliculosa*** | 0 | 0 | 85 (40.3) | 0 | 82 (40.6) | 60 (18.2) | 227 (29.0) |
| ***C. guilliermondii*** | 0 | 16 (55.2) | 37 (17.5) | 1 (20) | 37 (18.3) | 96 (29.2) | 187 (23.9) |
| ***C. lusitaniae*** | 3 (42.9) | 6 (20.7) | 51 (24.2) | 0 | 12 (5.9) | 17 (5.2) | 89 (11.4) |
| ***C. haenulonii*** | 0 | 0 | 0 | 0 | 0 | 85 (25.8) | 85 (10.9) |
| ***C. utilis*** | 0 | 0 | 23 (10.9) | 0 | 34 (16.8) | 0 | 57 (7.3) |
| ***C. famata*** | 2 (28.6) | 2 (6.9) | 0 | 0 | 2 (1.0) | 37 (11.2) | 43 (5.5) |
| ***C. Rugosa*** | 0 | 1 (3.4) | 4 (1.9) | 0 | 10 (5.0) | 0 | 15 (1.9) |
| ***C. dubliniensis*** | 1 (14.3) | 0 | 0 | 0 | 6 (3.0) | 4 (1.2) | 11 (1.4) |
| ***C. sake*** | 0 | 0 | 0 | 2 (40) | 0 | 9 (2.7) | 11 (1.4) |
| ***C. blankii*** | 0 | 0 | 0 | 0 | 10 (5.0) | 0 | 10 (1.3) |
| ***C. orthopsilosis*** | 0 | 0 | 5 (2.4) | 0 | 3 (1.5) | 0 | 8 (1.0) |
| ***C. kefyr*** | 0 | 0 | 2 (0.9) | 1 (20) | 2 (1.0) | 2 (0.6) | 7 (0.9) |
| ***C. stellatoidea*** | 0 | 0 | 0 | 0 | 0 | 6 (1.8) | 6 (0.8) |
| ***C. humicola*** | 0 | 1 (3.4) | 1 (0.5) | 0 | 0 | 3 (0.9) | 5 (0.6) |
| ***C. lipolytica*** | 0 | 2 (6.9) | 0 | 1 (20) | 1 (0.5) | 1 (0.3) | 5 (0.6) |
| ***C. intermedia*** | 0 | 1 (3.4) | 0 | 0 | 0 | 4 (1.2) | 5 (0.6) |
| ***C. albicans mutants*** | 0 | 0 | 0 | 0 | 0 | 4 (1.2) | 4 (0.5) |
| ***C. fabianii*** | 0 | 0 | 3 (1.4) | 0 | 0 | 0 | 3 (0.4) |
| ***C. magnoliae*** | 1 (14.3) | 0 | 0 | 0 | 1 (0.5) | 0 | 2 (0.3) |
| ***C. inconspicua*** | 0 | 0 | 0 | 0 | 1 (0.5) | 0 | 1 (0.1) |
| ***C. lambica*** | 0 | 0 | 0 | 0 | 1 (0.5) | 0 | 1 (0.1) |
| ***C. cerevisiae*** | 0 | 0 | 0 | 0 | 0 | 1 (0.3) | 1 (0.1) |
| **Total isolates^§^** | 7 | 29 | 211 | 5 | 202 | 329 | 783 |

^§^A total of 491 isolates were either mixed species or non-specified *Candida spp*.

Table S5 The susceptibility of NIC isolates.

|  | Fluconazole | Voriconazole | Itraconazole | Micafungin | Caspofungin | Amphotericin B |
| --- | --- | --- | --- | --- | --- | --- |
| ***C. albicans, number of resistant isolates (%), total isolates*** | | | | | | |
| African | 5 (0.8), 603 | 0, 60 | 0, 44 | 0, 44 | 0, 56 | 0, 12 |
| Americas | 1 (1.6), 61 | 0, 3 | 0, 12 | 0, 3 | 0, 3 | 0, 12 |
| Eastern Mediterranean | 2 (1.2), 172 | 0, 169 | 4 (4.8), 83 | 0, 8 | 0, 8 | 1 (1.3), 80 |
| European | 1 (14.3), 7 | NA | NA | NA | NA | 0, 7 |
| South-East Asia | 48 (25.7), 187 | 1 (0.4). 145 | 0, 75 | 1 (1.3), 80 | 2 (2.0), 101 | 8 (3.6), 220 |
| Western Pacific | 35 (6.2), 562 | 1 (0.5), 201 | 8 (3.1), 259 | 0, 5 | 0 | 3 (0.3), 889 |
| Total | 92 (5.8), 1592 | 2 (0.3), 578 | 12 (2.5), 473 | 1 (0.7), 140 | 2 (1.2), 168 | 12 (1.0), 1220 |
| ***C. parapsilosis, number of resistant isolates (%), total isolates*** | | | | | | |
| African | 624 (57.8), 1079 | 44 (17.1), 257 | 47 (20.9), 225 | 0, 225 | 0, 252 | 0, 27 |
| Americas | 0, 49 | 0, 12 | 0, 21 | 0, 12 | 0, 12 | 0, 21 |
| Eastern Mediterranean | 2 (1.6), 128 | 0, 126 | 0, 20 | NA | NA | 0, 18 |
| European | 0, 4 | NA | NA | NA | NA | 0, 4 |
| South-East Asia | 21 (21.0), 100 | 2 (1.5), 92 | 0, 31 | 0, 17 | 0, 29 | 4 (3.7), 108 |
| Western Pacific | 20 (7.8), 256 | 48 (30.4), 158 | 1 (0.8), 129 | NA | 0, 20 | 0, 373 |
| Total | 667 (41.3), 1616 | 94 (14.6), 645 | 48 (11.3), 426 | 0, 254 | 0, 313 | 4 (0.7), 551 |
| ***C. tropicalis, number of resistant isolates (%), total isolates*** | | | | | | |
| African | 1 (3.8), 26 | 0, 5 | NA | NA | NA | NA |
| Americas | 0, 19 | NA | 0, 4 | NA | NA | 0, 4 |
| Eastern Mediterranean | 2 (0.7), 288 | 1 (0.3), 288 | 0, 8 | 0, 3 | 0, 3 | 0, 8 |
| European | NA | NA | NA | NA | NA | NA |
| South-East Asia | 83 (26.5), 313 | 1 (0.4), 243 | 10 (8.1), 124 | 0, 93 | 3 (2.1), 141 | 17 (7.1), 239 |
| Western Pacific | 8 (9.9), 81 | 0, 55 | 1 (1.6), 61 | NA | 0, 22 | 0, 113 |
| Total | 94 (12.9), 737 | 2 (0.3), 591 | 11 (5.6), 197 | 0, 96 | 3 (1.8), 166 | 17 (4.7), 364 |
| ***C. glabrata, number of resistant isolates (%), total isolates*** | | | | | | |
| African | 0, 98 | 0, 10 | 0, 8 | 0, 8 | 0, 9 | 0, 1 |
| Americas | 1 (25.0), 4 | NA | NA | NA | NA | NA |
| Eastern Mediterranean | 3 (37.5), 8 | 2 (25.0), 8 | 1 (25.0), 4 | 0, 2 | 0, 2 | 0, 4 |
| European | NA | NA | NA | NA | NA | NA |
| South-East Asia | 9 (15.5), 58 | 0, 31 | 0, 30 | 1 (9.1), 11 | 1 (5.9), 17 | 4 (5.8), 69 |
| Western Pacific | 24 (15.6), 111 | 2 (15.8), 45 | 6 (15.8), 38 | 0 | NA | 0, 85 |
| Total | 37 (13.3), 279 | 4 (8.8), 94 | 7 (8.8), 80 | 1 (4.8), 21 | 1 (3.6), 28 | 4 (2.5), 159 |
| ***C krusei, number of resistant isolates (%), total isolates*** | | | | | | |
| African | 89 (93.7), 95 | NA | NA | NA | NA | NA |
| Americas | 1 (20.0), 5 | NA | 0, 1 | NA | NA | 0, 1 |
| Eastern Mediterranean | 0, 12 | 1 (8.3), 12 | NA | NA | NA | NA |
| European | NA | NA | NA | NA | NA | NA |
| South-East Asia | 111 (70.3), 158 | 45 (30.4), 148 | 1 (1.0), 96 | 0, 72 | 7 (8.2), 85 | 13 (7.9), 165 |
| Western Pacific | 2 (18.2), 11 | 0, 6 | 0, 3 | NA | 0 | 0, 67 |
| Total | 203 (72.2), 281 | 46 (27.7), 166 | 1 (1.0), 100 | 0, 72 | 7 (8.2), 85 | 13 (5.6), 233 |
| ***C. auris, number of resistant isolates (%), total isolates*** | | | | | | |
| African | 0, 7 | NA | NA | NA | NA | NA |
| Americas | 13 (81.3), 16 | 12 (100), 12 | NA | 0, 4 | 0, 4 | 9 (56.3), 16 |
| Eastern Mediterranean | NA | NA | NA | NA | NA | NA |
| European | NA | NA | NA | NA | NA | NA |
| South-East Asia | 22 (84.6), 26 | 5 (19.2), 26 | 0, 9 | 0, 23 | 0, 6 | 0, 23 |
| Western Pacific | NA | NA | NA | NA | NA | NA |
| Total | 35 (71.4), 49 | 17 (44.7), 38 | 0, 9 | 0, 27 | 0, 10 | 9 (23.1), 39 |

Note: The NA referred to not applicable, indicating no data available for the denominator and numerator. The value zero revealed no resistant isolates identified in this systematic review

Table S6 Antifungal use by geographical location

| Geographic regions | Overall neonates | Fluconazole | Amphotericin B | Fluconazole with amphotericin B | Echinocandins (micafungin or caspofungin) | Others |
| --- | --- | --- | --- | --- | --- | --- |
| Afrian | 885 | 204 (23.1) | 371 (41.9) | 300 (33.9) | 0 | 10 (11.3) |
| Eastern Mediterranean | 89 | 32 (36.0) | 44 (49.4) | 9 (10.1) | 0 | 4 (4.5) |
| Europe | 238 | 137 (57.6) | 63 (26.5) | 34 (14.3) | 0 | 4 (1.7) |
| Latin America | 292 | 37 (12.7) | 192 (65.4) | 16 (5.5) | 13 (4.5) | 34 (11.6) |
| Southeast Asia | 237 | 135 (56.7) | 69 (29.1) | 6 (2.5) | 0 | 27 (11.4) |
| Western Pacific | 1,085 | 1,022 (94.2) | 28 (2.6) | 13 (1.2) | 8 (0.7) | 14 (1.3) |
